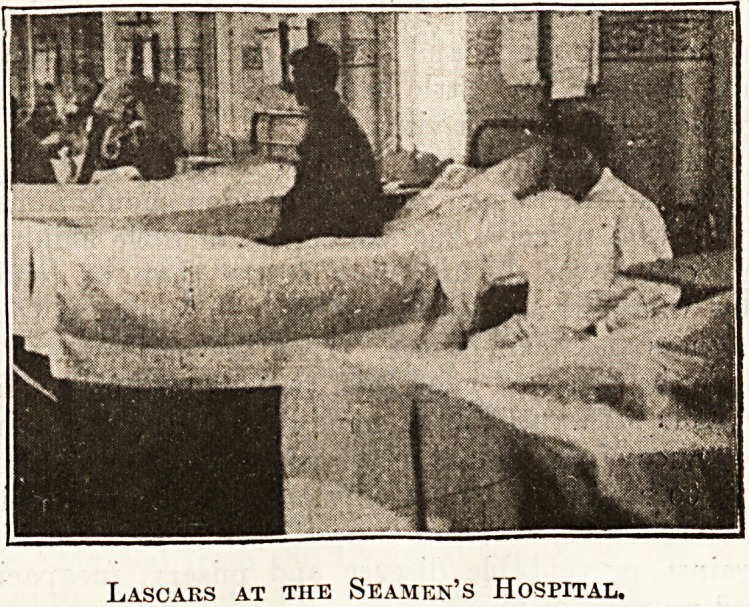# The Evolution of the Voluntary Hospital

**Published:** 1923-05

**Authors:** 


### EVOLUTION OF THE VOLUNTARY HOSPITAL.

### LECTURE BY MR. GODFREY HAMILTON.

ON the opening day Mr. Godfrey H. Hamilton, Secretary of the National Hospital for the Paralysed and Epileptic, Queen Square, Bloomsbury, gave a lecture on "The Evolution of the Voluntary Hospital," with cinematograph illustrations. The history of hospitals, said Mr. Hamilton, was associated

Microscopic Examination: Pathological Laboratory, National Hospital for the Paralysed and Epileptic.

with the history of organised treatment of the sick, of which the earliest records were to be found in connection with the monuments of ancient Egypt. Of the temple at Philae a part is dedicated to I-Em-Hetep, the Egyptian god of medicine, and there was little doubt that to this place the sick went for healing. Some interesting prescriptions taken from hieroglyphics and from the Bible were quoted, including the following Egyptian lotion for eyes: Myrrh, oxide of copper, citron pips and antimony, all mixed with pure white oil.

### How to Clear a Hospital Ward.

Mr. Hamilton went on to trace the treatment of the sick through ancient Greece and Rome until the foundation, after the Christian era, of hospitals in some way similar to those known to us to-day, the first of which were at Jerusalem and Constantinople. Proceeding to the Middle Ages, the foundations of the Hôtel Dieu at Paris and of the early hospitals in this country were detailed. It was mentioned that as late as 1700 the patients in the Hôtel Dieu were at times of pressure placed two and three in a bed. The early English hospitals were infirmaries connected with the monasteries, and to them resorted not only acute cases, but the chronic and destitute, the religious houses taking the place of the Poor Law, educational and hospital authorities. A story was told of a mediæval hospital in Italy where, the cases being mostly of a chronic nature, the patron of the hospital, a neighbouring nobleman, desired to clear the beds and make use of them for more hopeful cases. One of his friends dressed himself up in an imposing gown and hat, went to the hospital and gravely examined the patients, telling them that they were suffering from an acute skin disease which could be cured only by the application of a rare grease made by boiling down a human body. He suggested that it would be a good thing if one of the patients sacrificed himself for the rest, and promised to return in the evening to learn who had been chosen for the purpose. Upon his return the pseudo-physician was not surprised to find the place entirely deserted.

### 'Tis Fifty Years Since.

The lecturer dealt with the condition of hospitals in the middle of the nineteenth century and dwelt upon the great advantages that had accrued to these institutions through the work of Florence Nightingale and Lister. He quoted from the recent book by Sir Frederick Treves, "The Elephant Man and Other Stories," in which that distinguished practitioner draws a picture of the casualty department of the London Hospital fifty years ago, and asked the audience to compare it with the cinematograph pictures of present-day work. At the period referred to there was no ambulance service in London, and patients were either carried after injury by their legs and shoulders or placed upon a shutter. In the casualty department they were received by the house surgeon, who dealt with them, assisted by dressers and an untrained nurse. The patient, Sir Frederick Treves said, was examined on a leather couch saturated with the filth of ages. Mr. Hamilton pointed out what a large part beer played in mid-Victorian times, over 10 per cent. of the expenditure at King's College Hospital being in respect of it, while at St. Thomas's Hospital a brewery was installed in the building and the beer was served together with bread at midday with considerable ritual.

### How It Is Done.

At this point of the lecture the first series of pictures was shown portraying the handling of an accident case, its conveyance to the Middlesex

Finsen Ray Treatment, London Hospital.

Hospital and treatment in the casualty department. An accident to the eye was next shown as dealt with at the Royal Westminster Ophthalmic Hospital, where a steel splinter was extracted by means of the electro-magnet. Other parts of the film showed the out-patient department at St. Bartholomew's Hospital, the dispensary at the same hospital and at King's College Hospital, the taking of radiographs at the London Hospital and the Westminster Ophthalmic Hospital. The most interesting part of the film dealt with the treatment of foreign patients, Lascars, &c., at the Seamen's Hospital, Albert Dock. Mr. Hamilton dealt next with surgical cleanliness in operating theatres and wards, and again referred to the conditions of theatres and wards fifty years ago, quoting from Sir Frederick Treves's recent work, already mentioned. The general conditions, of surgical work were thus described :—

Taking a Radiograph at the London Hospital.

"There was no object in being clean. Indeed cleanliness was out of place. It was considered to be finicking and affected An executioner might as well manicure his nails before chopping off a head. The surgeon operated in a slaughterhouse-suggesting coat of black cloth. It was stiff with the blood and filth of years. The more sodden it was the more forcibly did it bear evidence to the surgeon's prowess. I, of course, commenced my surgical career in such a coat, of which I was quite proud. Wounds were dressed with 'charpe' soaked in oil. Both oil and dressing were frankly and exultingly septic—'charpe' was a species of cotton waste obtained from cast linen. It would probably now be discarded by a motor mechanic as being too dirty for use on a car. Owing to the suppurating wounds the stench in the wards was of a kind not easily forgotten."

The second series of pictures included a general view of a ward in King's College Hospital, showing the Teale Company's central stove, and the windows fitted with Rhodes patent sash hangings; a clever appliance for keeping water bottles hot was also demonstrated. The kitchen arrangements of King's College Hospital at a busy time of the day were shown, and the lecturer dealt with the great desirability of the serving of meals as hot as it is possible to make them. He further spoke of the desirability of simplicity in all hospital arrangements, advocating doors without ledges, rounded corners to the walls of all wards and rooms, hard plaster walls and teak or other hard wood flooring. The third series of pictures began with an interesting view of the pathological department at Queen Square, and a series of pictures showing the microbes of Asiatic fever, syphilis and relapsing fever, and an interesting film of microbes as seen under the microscope in diseased tissue. Further portions of the film shewed the Finsen ray department at the London Hospital with the active treatment for lupus going on. An operating theatre at the same hospital was also shewn with the administration of the anæsthetic, the surgeon and assistants at work and the movable observation gallery for the students. An animated picture of the well-known quadrangle at St. Bartholomew's Hospital, across which 300 nurses and 500 students pass every day, was displayed, as was the Exercise Department at King's College Hospital, with the patients being exercised under instruction.

After the third film, Mr. Hamilton showed a number of slides of modern hospital apparatus, including Manlove, Alliott & Co.'s sterilisers, the cooking apparatus of the Carron Co. and Messrs. James Slater & Co., useful hospital radiators, also by Slater's, and an automatic telephone exchange of the Relay Automatic Telephone Co. The lecturer closed with an interesting picture of the London Hospital from the air. Mr. Hamilton said that the outward appearance of the London Hospital, as seen from the Whitechapel Road, did not differ from the hospital as he had known it forty years ago, or from old prints in his possession of a much earlier date, yet within the hospital was constantly going on a scheme of renewal, remodelling, perfecting and enlarging. He suggested that this was typical of the voluntary system. Outwardly to the public the system was the same, but, while retaining its great principles in striving to alleviate pain and sickness, inwardly there was always going on a process of renewal, remodelling, perfecting and enlarging.

In King's College Hospital Kitchen.

We regret that space does not permit of the reproduction of the photograph of the London Hospital from the air, or of many of the other pictures which added so greatly to the interest of a lecture that was singularly revealing to such of the visitors as were not familiar with modern hospital methods. The improvements in hospital planning, practice and domestic management are now so rapid that these matters have become very much akin to a science. The days of the amateur are over.

Microtone Work, National Hospital.

Operating Theatre, London Hospital: Administering an Anæsthetic.

The films illustrating the lecture, which we here reproduce, were photographed and produced by Mr. Joseph Best, B.Sc. (London), of 32 Charing Cross, W.C.1, whose long specialisation in educational and scientific filming entitles him to be regarded as one of the chief authorities in that sphere. As director of the Educational Department of Pathé Freres, he produced most of their bacteriological and medical films. Since then, during the last ten years, he has accomplished notable work of a similar character on behalf of industrial and educational bodies and for scientific research workers and medical men. During the influenza epidemic Mr. Best was commissioned by the Government to produce a propaganda film showing the methods of infection and of combating the disease. This valuable film was officially circulated to the medical authorities throughout the country. One of Mr. Best's most highly technical films illustrated "Normal Labour," and showed the process of labour both from natural subjects and animated diagrams. For these diagrams more than 1,000 drawings were made. This film, of which copies can be had, was produced for Dr. Drummond Robinson, Obstetric Surgeon to the Westminster Hospital, and was shown to the Royal Society of Medicine in 1920. Notwithstanding the progress that has been made in this direction of late, the use of the cinema in medicine and surgery and for scientific purposes generally is obviously as yet in its infancy. The scope of the invention, and the improvements that will undoubtedly be made in it, should enable it before long to be in a very real sense a handmaid of service.

Removing a Steel Splinter by the Electro-Magnet.

Student's Gallery, London Hospital Theatre.

Lascars at the Seamen's Hospital.

## Figures and Tables

**Figure f1:**
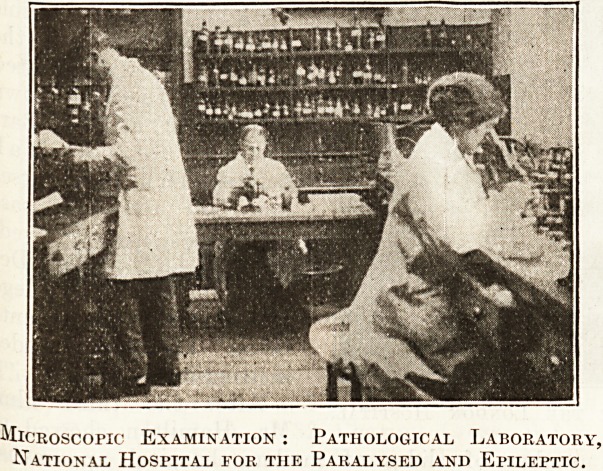


**Figure f2:**
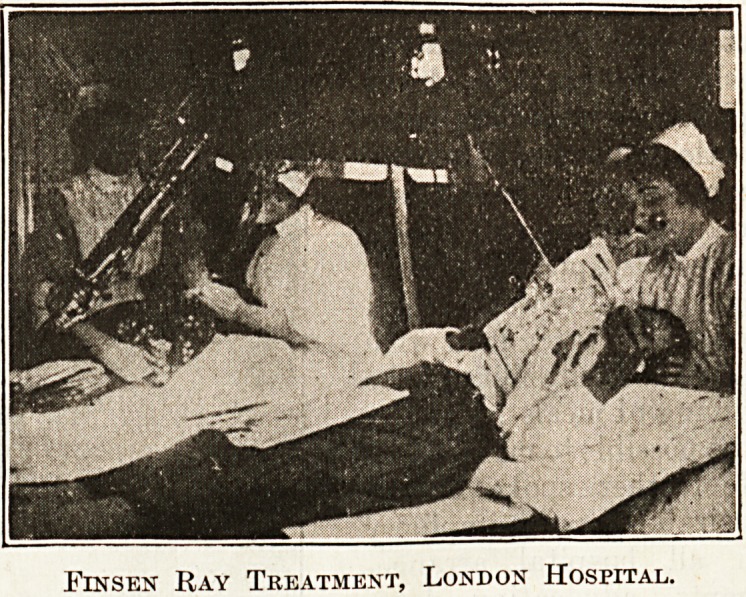


**Figure f3:**
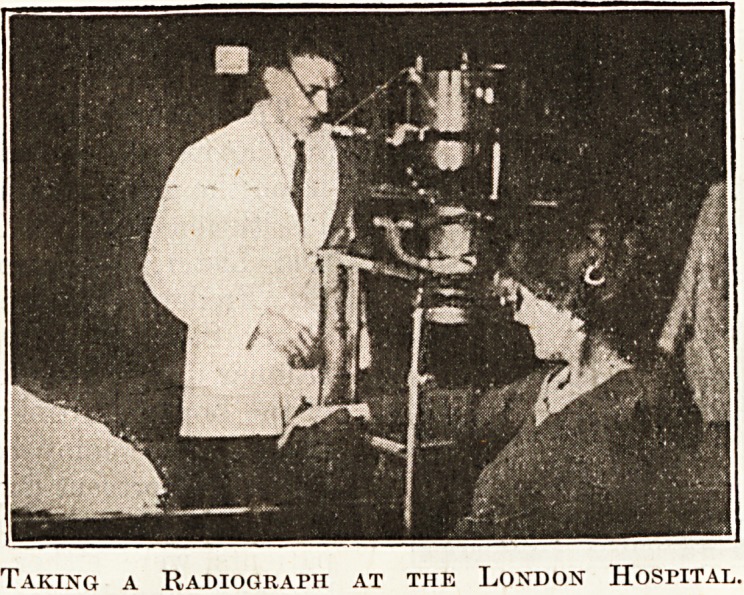


**Figure f4:**
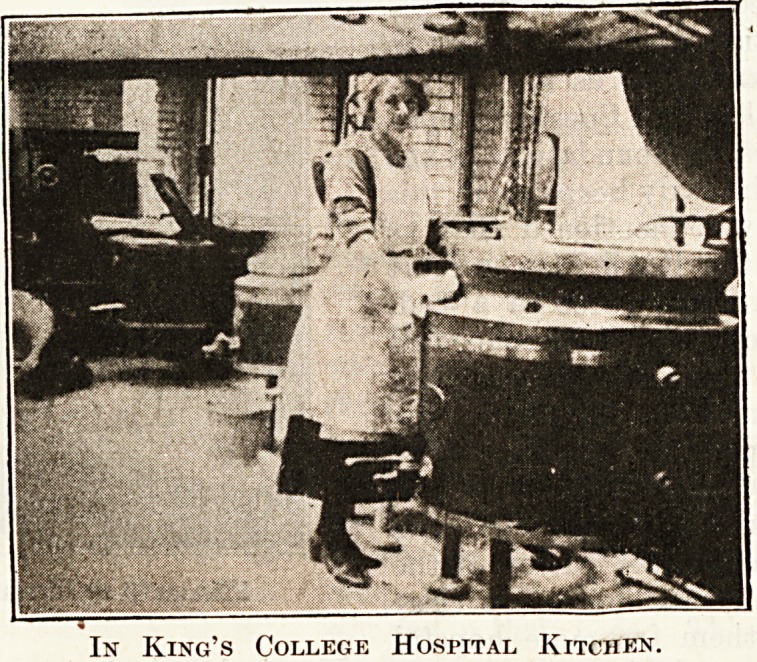


**Figure f5:**
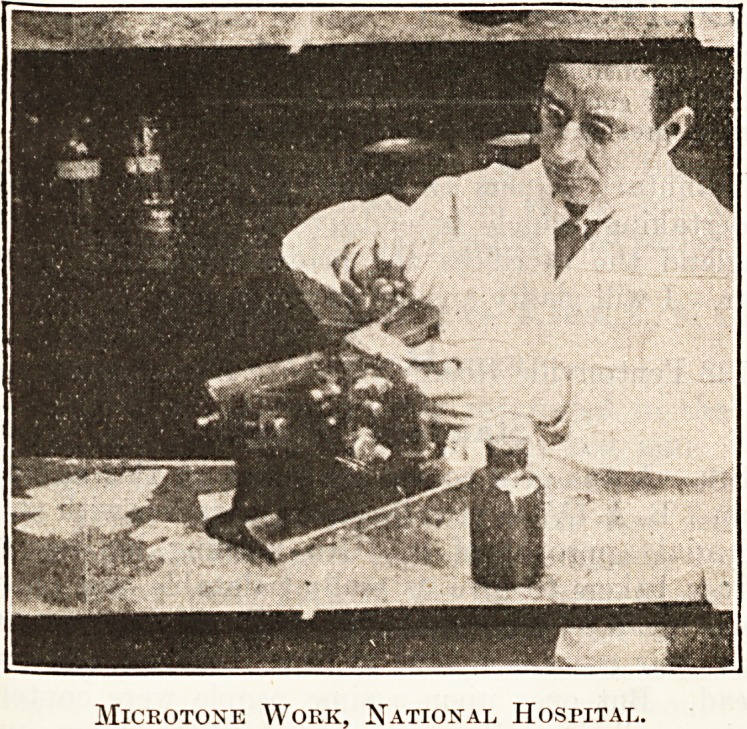


**Figure f6:**
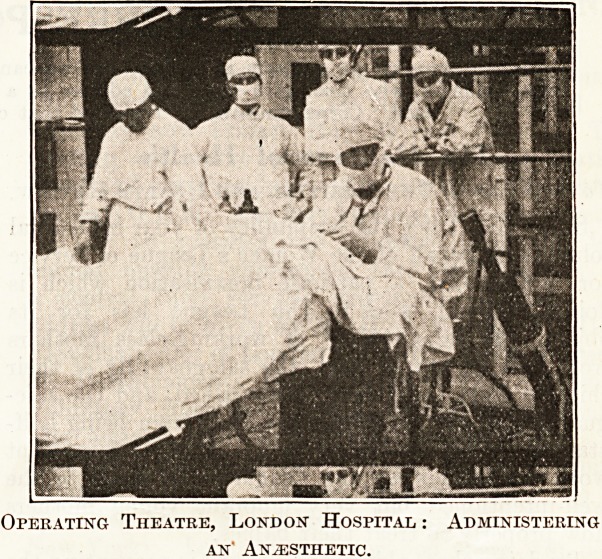


**Figure f7:**
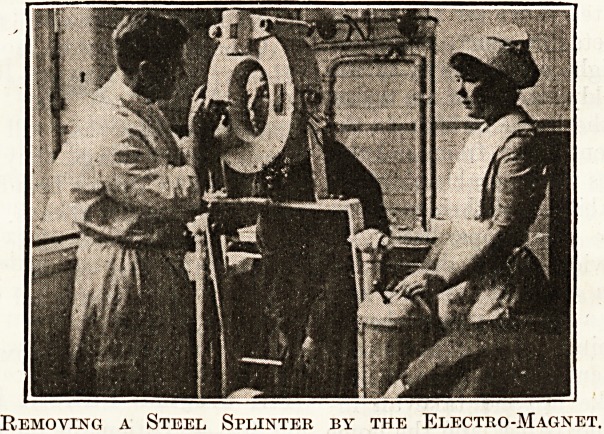


**Figure f8:**
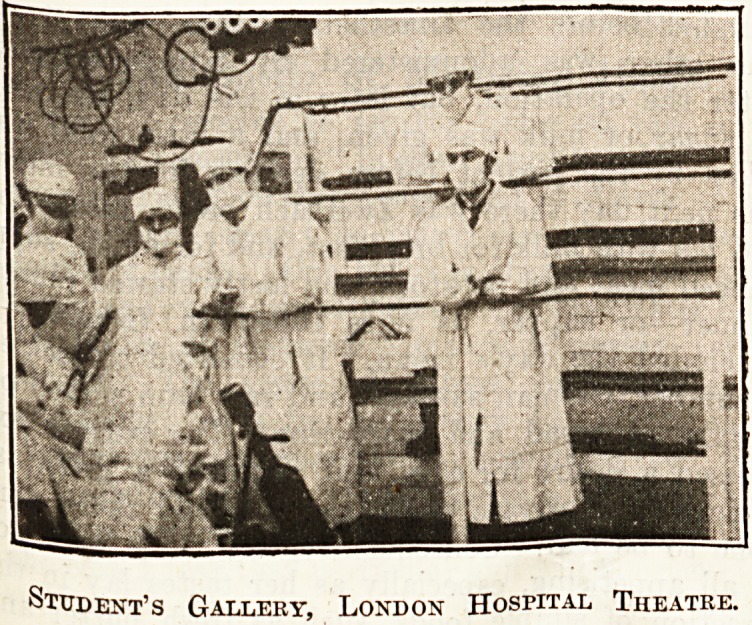


**Figure f9:**